# Effects of Marine Ranching on Phytoplankton Community: A Case Study in the Bailong Pearl Bay National Marine Ranching Demonstration Zone, China

**DOI:** 10.3390/biology15060477

**Published:** 2026-03-16

**Authors:** Jian Qin, Yu Guo, Chuanxin Qin, Gang Yu, Jinhui Sun, Karsoon Tan

**Affiliations:** 1 Tianjin Key Laboratory of Aqua-Ecology and Aquaculture, College of Fisheries, Tianjin Agricultural University, Tianjin 300384, China; 13853402266@163.com; 2 Key Laboratory of Marine Ranching, Ministry of Agriculture and Rural Affairs of China, South China Sea Fisheries Research Institute, Chinese Academy of Fishery Sciences, Guangzhou 510300, China; yuguo@scsfri.ac.cn (Y.G.); qincx@scsfri.ac.cn (C.Q.); yugang@scsfri.ac.cn (G.Y.); 3 National Agricultural Experimental Station for Fishery Resources and Environment Dapeng, Shenzhen 518121, China; 4 Key Laboratory of Efficient Utilization and Processing of Marine Fishery Resources of Hainan Province, Sanya Tropical Fisheries Research Institute, Sanya 572018, China; 5 Guangxi Key Laboratory of Beibu Gulf Biodiversity Conservation, Beibu Gulf Ocean Development Research Center, College of Marine Science, Beibu Gulf University, Qinzhou 535011, China

**Keywords:** marine ranching, phytoplankton, abundance, community structure, harmful algae blooms

## Abstract

Marine ranching is an important strategy for restoring marine habitats and replenishing aquatic populations. However, the effects of marine ranching on phytoplankton dynamics remain unclear. In this context, this study takes the Bailong Pearl Bay National Marine Ranching Demonstration Zone as an example to evaluate the influence of marine ranching on the spatial and temporal variation in phytoplankton abundance and community structure. A total of 101 phytoplankton species, spanning 44 genera and 26 families, were documented in the Bailong Pearl Bay National Marine Ranching Demonstration Zone, with 19 of these species identified as harmful algal blooms (HABs) or potential HABs. In spring, phytoplankton abundance remained relatively uniform across sampling stations, with community structure characterized by varying combinations of co-dominant species. In summer, phytoplankton density within the demonstration zone was higher than in adjacent regions. In contrast, lower phytoplankton abundance was observed within the demonstration zone during autumn and winter, periods marked by phytoplankton blooms in surrounding areas (autumn: *Chaetoceros lorenzianus*, *Rhizosolenia alata*, and *Skeletonema costatum*; winter: *Nitzschia pungens*). Correlation analysis indicated that phytoplankton abundance was positively correlated with nitrate and negatively correlated with phosphate, suggesting nutrient availability as a key driver of phytoplankton dynamics. These findings provide baseline information on how phytoplankton communities vary spatially and seasonally in relation to a marine ranching zone and offer insights to support the management and rehabilitation of marine ecosystems.

## Simple Summary

Scientists are still learning how marine ranching affects the tiny algae (phytoplankton) that form the base of the marine food web. This study investigated the Bailong Pearl Bay National Marine Ranching Demonstration Zone in China to find answers. Researchers discovered 101 different phytoplankton species in the area, including 19 types known to cause harmful algal blooms, or “red tides”. We found that during summer, the marine ranching area had more phytoplankton than the surrounding waters, showing that it helps these organisms thrive. However, during autumn and winter, when certain algae species began to bloom excessively, the ranching area actually had lower algae levels than other locations. This suggests that marine ranching not only boosts ocean productivity but may also naturally control harmful algal blooms. These findings are valuable for coastal communities and environmental managers because they demonstrate that well-designed marine ranching can help maintain healthy ocean ecosystems while potentially reducing the damaging effects of algal blooms on fisheries and tourism.

## 1. Introduction

Phytoplankton, the microscopic photosynthetic organisms that inhabit aquatic environments, play a pivotal role in marine ecosystems. As primary producers, they form the foundation of the marine food web, supporting a diverse array of organisms, from zooplankton to fish and marine mammals [1,2]. Beyond their ecological significance, phytoplankton contribute substantially to global biogeochemical cycles. However, some phytoplankton can cause harmful effects to aquatic life, human health, and the environment, known as harmful algae blooms (HABs) [3]. Their abundance, diversity, and distribution are influenced by various environmental factors, including nutrient availability, light conditions, temperature, and water circulation patterns [4]. Although there are several reports on the effects of marine ranching on chlorophyll-a [5] and biota (phytoplankton, zooplankton, and nekton) [6], detailed information on the influences of marine ranching on phytoplankton dynamics remains limited.

Marine ranching represents an innovative and eco-friendly approach to food production, leveraging the balance of marine ecosystems. By strategically deploying artificial reefs, enhancing biodiversity through controlled restocking, and utilizing resource replenishment techniques, it aims to restore marine habitats and replenish aquatic populations [7]. This integrated method fosters a synergistic relationship between ecological preservation and economic development, creating a balanced and sustainable system. Many countries have demonstrated that marine ranching is a powerful strategy for boosting fishery resources, ensuring their preservation and promoting sustainable utilization [8]. In China, the development of marine ranching began in 1979, and now at least 153 national marine ranching demonstration zones have been established and authorized [9,10].

Ecologically, the complex structure of artificial reefs provides shelter and feeding grounds for a wide variety of fish, including planktivorous species that prey on zooplankton [11,12]. A reduction in zooplankton population decreases the top-down pressure on phytoplankton and thus promotes the proliferation of phytoplankton in the water column around the reef [13]. In addition, artificial reefs enhance the recycling of nutrients not only from increased excretion of nutrients from fish [12], but the physical structure of the reef also disrupts currents, creating upwelling that brings nutrient-rich water from the bottom towards the surface, potentially stimulating phytoplankton growth [14,15].

In this context, it is important to conduct a systematic study to evaluate the influences of marine ranching on the spatial and seasonal abundance and community structure of phytoplankton. Based on environmental data, inorganic nutrient data, and phytoplankton data collected from the Bailong Pearl Bay National Marine Ranching Demonstration Zone and its surrounding areas during different seasons, this study not only provides insight into the effects of marine ranching on phytoplankton but also offers important guidance for the management and restoration of marine ecosystems.

## 2. Materials and Methods

### 2.1. Description of Study Area and Sample Collection

**Figure 1:**
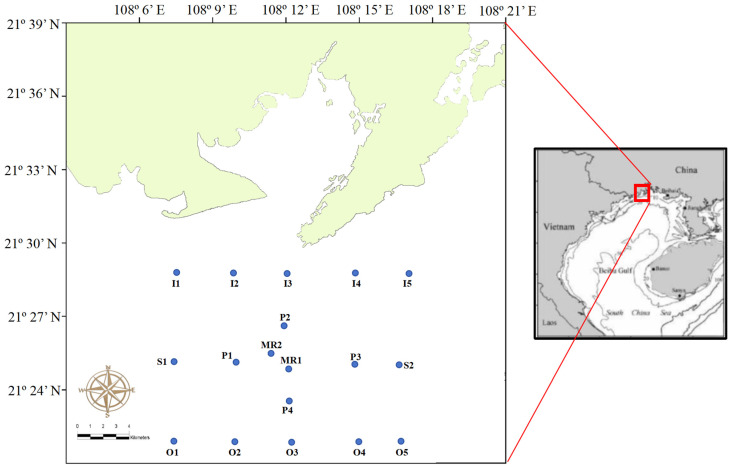
Sampling stations in the Bailong Pearl Bay National Marine Ranching Demonstration Zone and its surrounding areas. “MR” indicates marine ranching stations; “I” indicates inner shore stations; “S” indicates side stations with the same latitude as marine ranching areas; “P” indicates peripheral stations; and “O” indicates outer shore stations.

The Bailong Pearl Bay National Marine Ranching Demonstration Zone began in 2009, and by 2016, the area of 3.53 km^2^ was recognized as a National Marine Ranching Demonstration Zone [16]. According to China Ocean News [10], a total of 3328 artificial reefs and 6000 algal reefs have been constructed and deployed in the Bailong Pearl Bay National Marine Ranching Demonstration Zone. Additionally, 1.26 billion fish, shrimp, crabs, shellfish, and other organisms have been released for stock enhancement. In coastal and inner shore areas, over 333 hectares of shellfish culture rafts and 763 deep-water anti-typhoon fish aquaculture cages have been operated, with the production of marine economic fish and shellfish reaching 24,500 tons, worth 515 million RMB [10]. The Bailong Pearl Bay is characterized by a unique tropical and subtropical climate [17] and is sheltered from strong winds by the coastal area of Fangchenggang, which is rich in seagrass beds, coral reefs, and mangroves [18]. In this study, a total of 18 stations were established (Figure 1), including two stations within the Bailong Pearl Bay National Marine Ranching Demonstration Zone (marine ranching stations: MR1 and MR2; about 18 m depth), four stations located approximately 3 km away from the marine ranching area (peripheral stations: P1 to P4; about 20 m depth), two stations around 8 km away at the same latitude (side stations: S1 and S2; about 20 m depth), five stations in the inner shore areas (inner shore stations: I1 to I5; 10 to 15 m depth), and five stations in the outer shore areas (outer shore stations: O1 to O5; about 25 m depth).

Samplings were conducted in January 2020 (winter), September 2020 (summer), December 2020 (autumn), and April 2021 (spring), representing four different seasons. Environmental parameters, including water temperature, salinity, pH, dissolved oxygen, water depth (m), and visibility, were measured in situ by a multifunctional environmental sensor at 0.5 m below the water surface (YSI; Loveland, CO, USA), a depth sounder, and a Secchi disk, respectively. At each station, 2 L of seawater samples were collected at 0.5 m below the water surface using a water sampler. The samples were then divided into two 1 L aliquots. The first aliquot was filtered through a 0.45 μm GF/C membrane (Whatman) and stored at 0 °C for subsequent inorganic nutrient analysis. The second aliquot was fixed with Lugol’s solution for further species identification and cell counting. Sampling was conducted in triplicate.

### 2.2. Analysis for Inorganic Nutrients

The pre-filtered water samples were analyzed for inorganic nutrients using spectrophotometry, following the method described by the General Administration of Quality Supervision, Inspection and Quarantine of the People’s Republic of China (GB17378-2007) [19]. Specifically, the concentration of phosphate (PO_4_^3−^) was measured using the molybdenum blue method (GB17378.4/39.1-2007) at a wavelength of 880 nm with a detection limit of 0.05 μM (or 1.5 μg/L), nitrite (NO_2_^−^) was analyzed using the naphthylethylenediamine spectrophotometric method (GB17378.4/37.2-2007) at a wavelength of 543 nm with a detection limit of 0.02 μM (or 0.28 μg/L), nitrate (NO_3_^−^) was assessed via the cadmium column reduction method (GB17378.4/38.1-2007) at a wavelength of 554 nm with a detection limit of 0.1 μM (or 1.4 μg/L), and ammonium (NH_4_^+^) was quantified using the indophenol blue spectrophotometric method (GB17378.4/36.2-2007) at a wavelength of 640 nm with a detection limit of 0.1 μM (or 1.4 μg/L).

### 2.3. Analysis for Phytoplankton Abundance and Composition

In the laboratory, the seawater samples fixed with Lugol’s solution were concentrated to 50 mL using the Utermohl sedimentation method [20]. Phytoplankton identification was carried out to the species level following Cheng and Gao [21], Huang and Lin [22], Liu [23], Hartley [24], and Shu and Han [25]. Cell densities of phytoplankton were counted using a Sedwick–Rafter cell [26]. HABs and potential HABs were identified based on the IOC-UNESCO Taxonomic Reference List of Harmful Microalgae or their classification as toxic producers or bloom-forming species in previous studies [27].

### 2.4. Statistical Analysis

Statistical analysis was conducted using the IBM SPSS Statistics for Windows, Version 27.0, with significance set at *p* < 0.05. Before analysis, all variables were checked for normal distribution (Shapiro–Wilk test) and variance homogeneity (Levene’s test). If the assumptions of normality or homogeneity of variances were violated, non-parametric tests (e.g., Kruskal–Wallis, Spearman’s rank correlation) were used. Phytoplankton diversity was computed using the vegan package (Version 2.6-2) [28], assessed using the Shannon–Wiener index (H′) [29], and evenness was evaluated using Pielou’s evenness index (J′) [30] as follows:

*H*′ = −Σ (p_i_ × ln(n_i_/N))

where

Σ = the sum of the calculated values for all species.

n_i_ = number of individuals of a particular species.

N = total number of individuals of all species.

*J*′ = H′/ln(S)

where:

*H*′ = the observed Shannon–Wiener index;

S = total number of species.

One-way ANOVA with post hoc Tukey’s HSD test was applied separately to examine significant spatial or seasonal variations for each individual variable of interest, including environmental parameters, inorganic nutrients, phytoplankton abundance, the Shannon–Wiener index, and Pielou’s evenness index. Correlation coefficients were determined between key environmental variables and phytoplankton abundance using Spearman’s correlation. Principal Coordinates Analysis (PCoA) was performed using the R-language (V 4.5.1) vegan package [31] to examine the seasonal clustering of phytoplankton.

## 3. Results

### 3.1. Seasonal and Spatial Variation in Environmental Parameters

**Figure 2:**
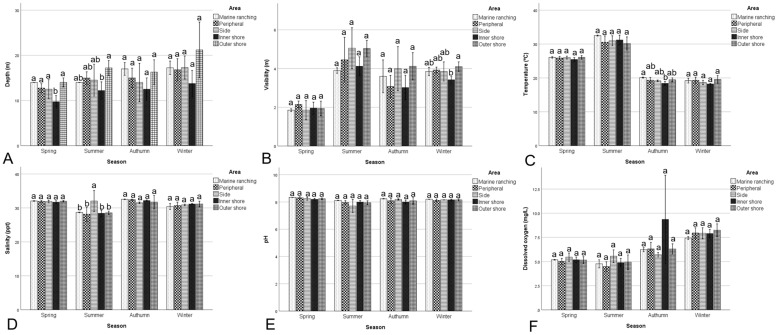
Environmental parameters ((**A**) depth, (**B**) visibility, (**C**) temperature, (**D**) salinity, (**E**) pH, and (**F**) dissolved oxygen) in the Bailong Pearl Bay National Marine Ranching Demonstration Zone and its sur-rounding areas. Different letters indicate significant differences (*p* < 0.05).

The seasonal and spatial variations in environmental parameters in the Bailong Pearl Bay National Marine Ranching Demonstration Zone and its surrounding areas are illustrated in Figure 2. No significant difference in water depth was observed (*p* > 0.05). The visibility of the seawater in summer (4.52 ± 0.78 m) was significantly higher (*p* < 0.05), while in spring (1.97 ± 0.29 m), it was significantly lower (*p* < 0.05) compared to other seasons (3.51 to 3.82 m) (*p* < 0.05). The water temperature showed a descending trend (*p* < 0.05) from summer (30.88 ± 1.62 °C), to spring (25.84 ± 0.50 °C), to autumn (19.16 ± 0.76 °C), and to winter (18.98 ± 0.91 °C). The salinity showed a descending trend (*p* < 0.05) from spring (31.87 ± 0.30 psu) and autumn (32.06 ± 1.02 psu) > winter (30.92 ± 0.69 psu) > summer (28.82 ± 1.72 psu). The pH in spring (8.25 ± 0.08) and winter (8.15 ± 0.07) was significantly higher (*p* < 0.05), while in summer (7.95 ± 0.19), it was significantly lower (*p* < 0.05) compared to other seasons. The DO in autumn (7.08 ± 2.69 mg/L) and winter (7.96 ± 0.52 mg/L) was significantly higher (*p* < 0.05) than in spring (5.17 ± 0.30 mg/L) and summer (4.87 ± 0.59 mg/L).

In spring, there were no significant differences (*p* > 0.05) in visibility, temperature, salinity, pH, and DO among sampling stations. In summer, the salinity in side stations was significantly higher (*p* < 0.05) than in other stations. In autumn, the temperature in inner shore stations was significantly lower (*p* < 0.05) than in marine ranching stations, while DO in inner shore stations was significantly higher (*p* < 0.05) than in other stations. In winter, no significant difference was observed in any environmental parameters between the marine ranching and other stations.

### 3.2. Seasonal and Spatial Variation in Inorganic Nutrients

**Figure 3:**
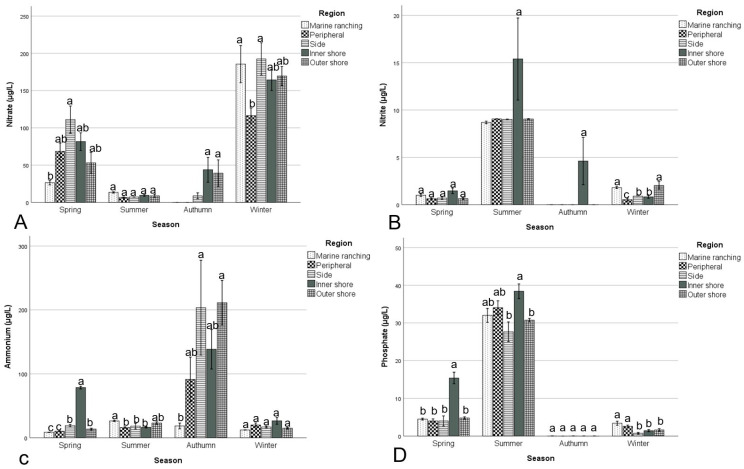
Inorganic nutrients ((**A**) nitrate, (**B**) nitrite, (**C**) ammonium, and (**D**) phosphate) in the Bailong Pearl Bay National Marine Ranching Demonstration Zone and its surrounding areas. Different letters indicate significant differences (*p* < 0.05).

The concentrations of inorganic nutrients in the Bailong Pearl Bay National Marine Ranching Demonstration Zone and its surrounding areas are illustrated in Figure 3. The nitrite concentration in summer (10.76 ± 9.10 μg/L) was significantly higher (*p* < 0.05) than in other seasons (0.91 to 1.28 μg/L). The nitrate concentration in winter (160.59 ± 54.67 μg/L) was significantly higher (*p* < 0.05), while in summer and autumn (8.85 to 24.03 μg/L), it was significantly lower (*p* < 0.05) compared to other seasons. The concentration of ammonium in autumn (142.03 ± 137.45 μg/L) was significantly higher (*p* < 0.05) than in other seasons (19.18 to 30.94 μg/L). The concentration of phosphate showed a descending trend from summer (33.39 ± 6.57 μg/L) > spring (7.44 ± 6.04 μg/L) > winter and autumn (0.005 to 1.88 μg/L).

In spring, there was no significant spatial variation (*p* > 0.05) in nitrite. However, nitrate in side stations was significantly higher (*p* < 0.05) than in marine ranching stations; ammonia in side and inner shore stations was significantly higher (*p* < 0.05) than in other stations, whereas phosphate in inner shore stations was significantly higher (*p* < 0.05) than in other stations. In summer, there were no significant differences (*p* > 0.05) in nitrate. However, the concentration of nitrite in inner shore stations was significantly higher (*p* < 0.05) than in other stations; the concentration of ammonia in marine ranching stations was significantly higher (*p* < 0.05) than in side, peripheral, and inner shore stations. In autumn, there were no significant differences (*p* > 0.05) in phosphate. The nitrate concentrations in inner shore and outer shore stations were significantly higher (*p* < 0.05) than in other stations; the nitrate concentrations in inner stations were significantly higher (*p* < 0.05) than in other stations, and the concentration of ammonia in side and outer shore stations was significantly higher (*p* < 0.05) than in marine ranching and peripheral stations. In winter, no significant difference (*p* > 0.05) was observed in ammonium concentration among areas, but the concentration of nitrite and nitrate in peripheral stations was significantly lower (*p* < 0.05) than in marine ranching stations, and the phosphate concentration in marine ranching and peripheral stations was significantly higher (*p* < 0.05) than in other stations.

### 3.3. Seasonal and Spatial Variation in Phytoplankton Abundance

**Figure 4:**
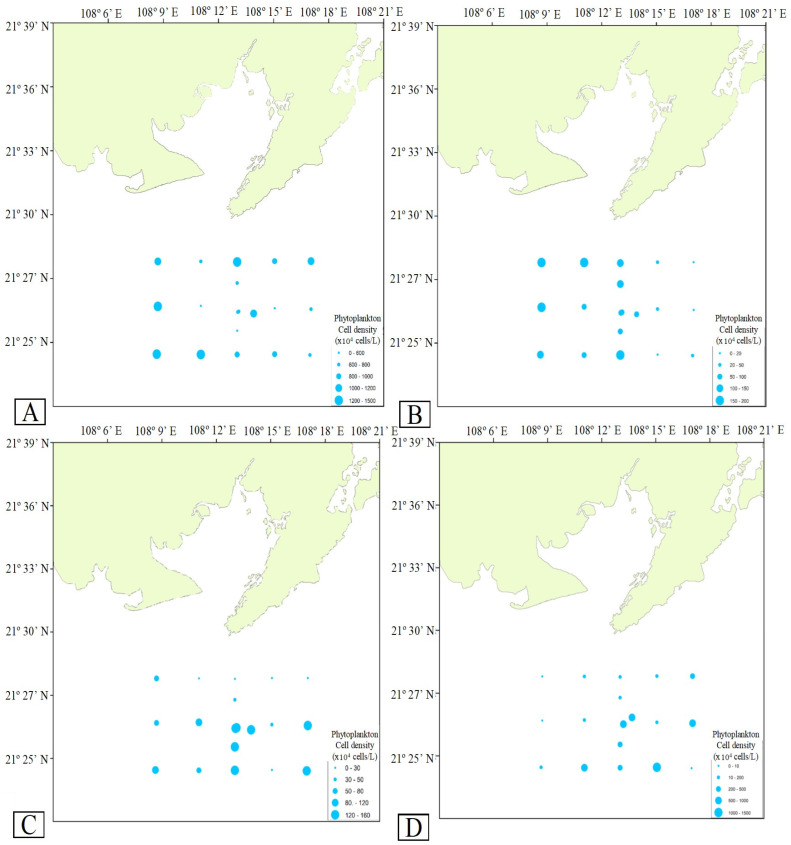
Phytoplankton abundance in the Bailong Pearl Bay National Marine Ranching Demonstration Zone and its surrounding areas during (**A**) spring, (**B**) summer, (**C**) autumn, and (**D**) winter.

The abundance of phytoplankton in the Bailong Pearl Bay National Marine Ranching Demonstration Zone and its surrounding areas is illustrated in Figure 4. The phytoplankton abundance showed a descending trend (*p* < 0.05) from spring (881.49 ± 69.09 × 10^4^ cells/L), to winter (328.60 ± 92.01 × 10^4^ cells/L), to summer (84.66 ± 13.23 × 10^4^ cells/L), and to autumn (15.50 ± 2.68 × 10^4^ cells/L).

There were no significant spatial variations (*p* > 0.05) in phytoplankton abundance during spring, but it was significantly higher (*p* < 0.05) in marine ranching stations (154.12 ±1.24 × 10^4^ cells/L) than in other stations (55.52 to 107.57 × 10^4^ cells/L) in summer. In autumn, the phytoplankton abundance in outer shore (24.87 ± 5.54 × 10^4^ cells/L) and peripheral (19.84 ± 3.79 × 10^4^ cells/L) stations was significantly higher (*p* < 0.05) than in marine ranching (5.12 ± 2.27 × 10^4^ cells/L) and inner shore (8.01 ± 4.27 × 10^4^ cells/L) stations. In winter, the abundance of phytoplankton in outer shore (622.49 ± 229.05 × 10^4^ cells/L) and side (455.84 ± 280.22 × 10^4^ cells/L) stations was significantly higher (*p* < 0.05) than in marine ranching stations (28.29 ± 21.24 × 10^4^ cells/L).

### 3.4. Seasonal and Spatial Variation in Phytoplankton Community Structure

The phytoplankton community structure in the Bailong Pearl Bay National Marine Ranching Demonstration Zone and its surrounding areas during spring, summer, autumn, and winter is summarized in Table 1, Table 2, Table 3 and Table 4 and Supplementary Tables S1–S4, respectively. A total of 101 phytoplankton species, belonging to 44 genera and 26 families, were identified. The Shannon–Wiener diversity index (*H*′) of phytoplankton in summer (3.28 ± 0.41) and autumn (3.92 ± 0.83) was significantly higher (*p* < 0.05) than in spring (2.58 ± 0.50) and winter (1.21 ± 0.81). The Pielou’s evenness index (*J*′) of phytoplankton in winter (0.27 ± 0.21) was significantly lower (*p* < 0.05) than in other seasons (0.66 ± 0.07 to 0.78 ± 0.10).

In spring, different stations had different combinations of co-dominant phytoplankton species, with the relative abundance of each co-dominant species generally around 10%. In summer, *Rhizosolenia hyalina* was the dominant species in MR1–2, P2–4, S2, I3–5, and O3–5, with a relative abundance of >20%. In autumn, *Chaetoceros lorenzianus*, *R. alata*, and *Skeletonema costatum* were the co-dominant species, with relative abundances of around 15 to 30% in most stations. In winter, *Nitzschia pungens* blooms occurred, especially in stations MR1–2, P2–P3, S2, I3, and O1–4, where the relative abundance of this species was >80%.

### 3.5. Occurrence and Distribution of HABs and Potential HABs

Among the phytoplankton, there were at least 19 species identified as HABs or potential HABs, including *Akashiwo sanguinea*, *Tripos furca*, *T. fusus*, *T. tripos*, *Chaetoceros affinis*, *Chaetoceros curvisetus*, *Dinophysis caudata*, *Nitzschia pungens*, *Noctiluca scintillans*, *Protoperidinium pellucidum*, *P. divergens*, *P. depressum*, *P. conicum*, *P. pentagonum*, *Prorocentrum micans*, *Prorocentrum sigmoides*, *S. costatum*, and *Trichodesmium* spp. There were no significant differences (*p* > 0.05) in the relative abundance of HABs and potential HABs among stations.

In spring and summer, HABs and potential HABs accounted for 15.36 ± 7.74% and 14.81 ± 7.95% of the total phytoplankton, respectively, with no dominant HABs or potential HABs documented. In autumn, HABs and potential HABs accounted for 21.89 ± 15.00% of the total phytoplankton, with *S. costatum* being dominant (13.15 ± 11.39% of the total phytoplankton). In winter, *Nitzschia pungens* bloomed in the study area, with a relative abundance of 82.46 ± 14.65%.

### 3.6. Relationship Between Environmental Parameters and Phytoplankton Abundance

Spearman’s correlation coefficients among environmental parameters, inorganic nutrients, and phytoplankton abundance are summarized in Table 5. The abundance of phytoplankton was positively correlated with temperature (*r* = 0.31; *p* < 0.05), pH (*r* = 0.31; *p* < 0.05), nitrite (*r* = 0.20; *p* < 0.05), nitrate (*r* = 0.40; *p* < 0.05), and phosphate (*r* = 0.37; *p* < 0.05), while it was negatively correlated with ammonia (*r* = −0.19; *p* < 0.05) and DO (*r* = −0.26; *p* < 0.05). The relative abundance of HABs and potential HABs was positively correlated with DO (*r* = 0.52; *p* < 0.05) and nitrate (*r* = 0.50; *p* < 0.05) but negatively correlated with temperature (*r* = −0.49; *p* < 0.05) and phosphate (*r* = −0.30; *p* < 0.05).

The concentration of nitrite was positively correlated with temperature (*r* = 0.60; *p* < 0.05) but negatively correlated with salinity (*r* = −0.66; *p* < 0.05), pH (*r* = −0.32; *p* < 0.05), and DO (*r* = −0.39; *p* < 0.05). The concentration of nitrate was positively correlated with DO (*r* = 0.34; *p* < 0.05) but negatively correlated with temperature (*r* = −0.28; *p* < 0.05). The concentration of phosphate was positively correlated with temperature (*r* = 0.80; *p* < 0.05) but negatively correlated with salinity (*r* = −0.62; *p* < 0.05), pH (*r* = −0.24; *p* < 0.05), and DO (*r* = −0.64; *p* < 0.05).

**Figure 5:**
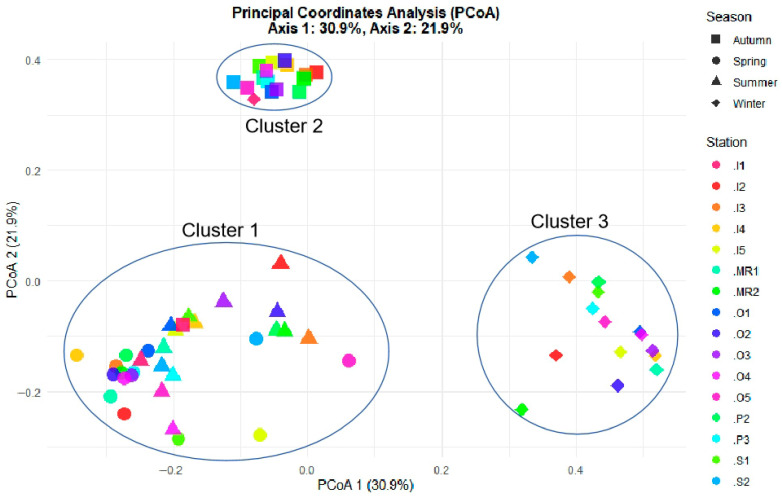
Principal coordinate analysis between environmental parameters and phytoplankton.

The results of PCoA revealed a strong seasonal variation (explaining 52.8% of the total variance) in phytoplankton community structure, where three distinct clusters were formed: cluster 1 represents samples collected from spring and summer, cluster 2 represents samples collected from autumn, and cluster 3 represents samples collected from winter (Figure 5).

## 4. Discussion

The Bailong Pearl Bay National Marine Ranching Demonstration Zone is a pioneering initiative in marine conservation and sustainable aquaculture [7]. Located in the coastal waters of Guangxi, China, this zone has been developed to restore marine ecosystems, enhance biodiversity, and promote eco-friendly fisheries [16]. Significant efforts have been made to establish artificial reefs, seagrass beds, and shellfish habitats, which provide breeding grounds for marine species and improve water quality [10]. The present study identified 101 phytoplankton species, categorized into 44 genera and 26 families. Among them, 19 species are HABs or potential HABs. These numbers are lower than the 147 phytoplankton species reported in an offshore station in the Beibu Gulf [32]; 205 phytoplankton species [3,33], including 38 HABs [34], were reported in the Maowei Sea, northern Beibu Gulf; and 180 phytoplankton species were reported in a large portion of Beibu Gulf [35]. This is not surprising, as the current study used conventional morphological and taxonomic identification, whereas He et al. [32] and Tan et al. [3] employed a metabarcoding approach that enables the identification of small phytoplankton species and those that are difficult to preserve. Therefore, a combination of key conventional morphological, taxonomic, and metabarcoding methods is recommended in future studies of phytoplankton community structure. However, the number of identified phytoplankton species in this study was higher than the 87 phytoplankton species reported in the southern Beibu Gulf, which was based on conventional microscopic observation methods [36]. The phytoplankton abundance reported in the present study (16 to 881 × 10^4^ cells/L) was comparable to that reported in a big portion of Beibu Gulf (27 to 824 × 10^4^ cells/L) [35] but higher than that in the southern Beibu Gulf (0.008 to 0.47 × 10^4^ cells/L) [36].

In spring, there were no significant differences in phytoplankton abundance, with different combinations of co-dominant phytoplankton species. This observation might indicate that the environment provides sufficient resources to support similar levels of phytoplankton biomass, irrespective of which species are dominant [37]. In summer, the abundance of phytoplankton in marine ranching and inner shore stations was significantly higher than in other stations. This might be attributed to artificial reefs amplifying turbulence in marine environments, promoting the upward movement of nutrients from deeper waters to surface layers, and promoting the growth and proliferation of phytoplankton [14,15]. Likewise, in the Uwa Strait, Japan, artificial reef structures measuring 95 m in length and 10 m in height were engineered to generate upwelling effects, resulting in a two- to three-fold rise in chlorophyll-a [38]. In fact, Kim and Shimasaki [39] suggest that artificial reef systems may play a critical role in nutrient replenishment during the summer months, when water stratification restricts vertical mixing, thereby confining nutrients to deeper layers. However, the possibility cannot be ruled out that the higher phytoplankton abundance in marine ranching and inner shore stations might also be caused by freshwater influx due to the summer rainy seasons in the Beibu Gulf [40]. In fact, studies utilizing remote sensing technology have revealed a strong correlation between worldwide phytoplankton blooms and precipitation patterns. These rainfall events contribute substantial freshwater influxes, delivering ample nutrients to coastal regions to support the growth and proliferation of phytoplankton [41].

In autumn, the abundance of phytoplankton in marine ranching stations was lower than in many other areas, especially in outer shore and peripheral stations. This observation could be attributed to the higher zooplankton abundance in marine ranching (1072.41 ± 381.19 ind/m^3^) compared to outer shore stations (325.74 ± 277.37 ind/m^3^) (unpublished data). In fact, many studies have shown that artificial reefs promote the proliferation and accumulation of zooplankton and bivalves by providing food and shelter [5,42,43]. For example, in the Luanhe River Estuary, the zooplankton biomass after reef construction (32.91 mg/m^3^) was significantly higher than before reef construction (2.72 mg/m^3^) [43]. Similarly, in Xiangshan Bay, China, the zooplankton abundance in artificial reef areas was 1.34-fold higher than in control areas [5]. *Chaetoceros lorenzianus*, *Rhizosolenia alata*, and *S. costatum* were the co-dominant phytoplankton species in autumn. Among them, *S. costatum* is a non-toxic HAB species that generates substantial biomass during blooms, which impedes light penetration. The subsequent decay of these algae releases harmful gases and unpleasant odors, posing risks to aquatic organisms and leading to significant financial setbacks for aquaculture [44]. For instance, in Lianyungang, China, annual *S. costatum* blooms have been reported [45], with a notable event in 2005 resulting in extensive fish deaths and an economic loss of 0.74 million USD [46]. Similarly, in Pangasinan, Philippines, *S. costatum* blooms in milkfish (*Chanos chanos*) farming areas during 2002 and 2010 led to fish die-offs valued at 9 million USD [47] and 1.1 million USD [48], respectively. Therefore, regular monitoring of this species should be conducted to minimize the negative impact of its blooms on ecosystems and the aquaculture industry.

Similarly, in winter, the abundance of phytoplankton in marine ranching stations was lower than in many other areas, especially in side and outer shore stations, despite the high nitrite, nitrate, and phosphate concentrations in marine ranching stations. The higher nutrient concentrations in marine ranching stations might be attributed to the upwelling induced by the turbulence created by bottom currents hitting artificial reef structures [14,15]. It is worth noting that *Nitzschia pungens*, a HAB species, bloomed in most stations, including marine ranching stations, during winter. *Nitzschia pungens* is a domoic acid producer that was first described in 1987 in Eastern Prince Edward Island, Canada, causing human poisoning after consuming *Mytilus edulis*, associated with at least three deaths and 107 cases of food poisoning [49]. *Nitzschia pungens* is found across nearly every continent worldwide, spanning Europe, the Americas, Asia, and Oceania [50]. In China, *Nitzschia pungens* has been frequently documented in coastal areas, such as Dalian, Qingdao, Xiamen, Hong Kong, and Zhanjiang [51]. *Nitzschia pungens* has been observed to create extensive blooms across numerous areas in China, such as the Bohai Sea, the Yellow Sea, the Changjiang Estuary, the East China Sea, and the South China Sea [52,53,54,55]. Therefore, special attention is required on the impact of this species on the ecosystem of the Bailong Pearl Bay National Marine Ranching Demonstration Zone in future studies.

The results of the correlation revealed that the abundance of phytoplankton and HABs in the Bailong Pearl Bay National Marine Ranching Demonstration Zone and its surrounding waters is mainly positively correlated with nitrate but negatively correlated with phosphate. These observations suggest that nitrate may be a key limiting nutrient for phytoplankton growth in this environment (mesotrophic water), which could result from upwelling or anthropogenic inputs like aquaculture, as the negative correlation with phosphate indicates it is unlikely to result from river runoff [56]. Therefore, controlling nitrate inputs from aquaculture may be more effective in managing phytoplankton blooms and HABs in the Bailong Pearl Bay National Marine Ranching Demonstration Zone and surrounding areas. Among monitoring methods, an integrated multitrophic aquaculture system could be an ideal approach to monitor nutrients, as some organisms, such as seaweed and bivalves, are known to effectively reduce nitrogen and phosphorus in water [7]. However, caution is warranted when interpreting correlation-based results, as they reflect associations rather than causative relationships and may be influenced by confounding factors such as seasonal variability, hydrodynamics, and differential nutrient uptake by phytoplankton. In spring and winter, N/P ratios ranged from 16:1 to 24:1, approximating or exceeding the Redfield ratio (16:1), suggesting potential phosphorus limitation or balanced availability during these periods. In contrast, summer and autumn were characterized by N/P ratios below 10:1, indicating potential nitrogen limitation, which aligns with the positive correlation between phytoplankton abundance and nitrate during these seasons. These seasonal shifts in N/P ratios likely reflect complex interactions between nutrient inputs, biological uptake, and physical mixing processes.

The findings of this study offer several critical insights for environmental management and marine policy formulation, particularly in the context of China’s expanding national marine ranching program. This study demonstrates that marine ranching actively modulates phytoplankton dynamics. The observation that phytoplankton abundance was significantly lower within the ranching zone during autumn and winter bloom periods (including blooms of *S. costatum* and *N. pungens*) suggests that artificial reef ecosystems may exert a natural top-down control on algal blooms. This implies that strategic deployment of marine ranching could serve as a nature-based solution for mitigating the intensity of HABs. However, caution is warranted, as the changes in environmental and biological factors before and after this test region was set up for marine ranching will provide additional useful information. Therefore, it is highly recommended to repeat this survey in five to ten years’ time to further evaluate the effects of marine ranching on phytoplankton community structure.

## 5. Conclusions

In conclusion, 101 phytoplankton species (44 genera and 26 families), including 19 species of HABs or potential HABs, were identified in the Bailong Pearl Bay National Marine Ranching Demonstration Zone and its surrounding waters. In spring, the phytoplankton abundance was consistent across stations, with community structure characterized by varying combinations of co-dominant species. In summer, higher phytoplankton abundance was observed within the Bailong Pearl Bay National Marine Ranching Demonstration Zone compared to surrounding areas. Conversely, during autumn and winter, phytoplankton abundance within the ranching zone was lower than in adjacent areas, coinciding with blooms of *Chaetoceros lorenzianus*, *Rhizosolenia alata*, and *Skeletonema costatum* in autumn and *Nitzschia pungens* in winter. Correlation analyses revealed that phytoplankton abundance was positively associated with nitrate and negatively associated with phosphate, highlighting the potential role of nutrient dynamics in shaping phytoplankton communities. These findings contribute to our understanding of spatial and seasonal phytoplankton variability in relation to marine ranching and provide a scientific basis for informed decision-making in marine ecosystem management and conservation.

## Figures and Tables

**Table 1:** Relative abundance (%) of the top 20 phytoplankton taxa in the Bailong Pearl Bay National Marine Ranching Demonstration Zone and its surrounding areas during spring.

	MR1	MR2	P1	P2	P3	P4	S1	S2	I1	I2	I3	I4	I5	O1	O2	O3	O4	O5
*Coscinodiscus granii*	10.10	4.30	4.59	13.48	6.27	0.72	13.09	3.97	24.64	7.20	8.81	18.18	1.83	6.30	8.24	12.70	0.00	3.39
*Thalassiosira subtilis*	2.07	4.57	0.31	7.54	3.51	0.00	2.80	11.86	2.32	8.90	3.14	9.09	71.34	9.33	2.61	0.00	0.00	11.75
*Bacteriastrum hyalinum*	10.63	12.99	12.98	11.30	27.15	0.00	3.20	10.54	5.51	3.39	11.32	0.00	0.00	4.64	7.21	4.01	0.00	11.32
*Thalassionema frauenfeldii*	11.15	9.56	7.29	14.93	10.65	24.01	1.30	8.93	3.48	0.00	2.52	0.00	1.22	4.20	4.52	5.19	16.98	9.44
*Chaetoceros coarctatus*	4.97	2.01	10.59	2.75	3.59	4.66	23.28	0.00	12.17	20.34	16.35	0.00	0.00	16.50	7.77	3.34	0.00	4.67
*Chaetoceros lorenzianus*	6.33	9.66	8.39	4.06	3.68	5.02	5.29	5.86	10.72	30.93	5.66	0.00	2.44	5.13	7.37	5.31	1.89	8.50
*Stephanopyxis palmeriana*	4.79	3.50	0.37	4.78	4.39	1.43	2.10	11.01	4.06	0.00	8.18	0.00	3.66	1.67	3.41	12.89	0.00	6.41
*Guinardia flaccida*	4.63	8.62	7.84	1.16	3.88	11.47	1.50	3.97	0.00	1.27	1.26	0.00	0.00	0.93	3.01	3.11	16.04	1.28
*Skeletonema costatum*	3.32	2.36	2.94	1.88	4.72	6.45	4.80	0.95	0.00	0.00	18.87	0.00	0.00	3.58	2.22	3.46	6.60	4.67
*Hemiaulus sinensis*	4.41	12.09	4.65	4.20	4.09	0.00	2.30	1.84	0.00	0.85	2.52	0.00	0.00	2.35	9.43	9.39	0.00	4.43
*Rhizosolenia hyalina*	4.45	3.60	2.76	2.75	2.97	2.87	3.40	4.30	1.74	0.00	0.00	0.00	0.00	0.87	7.29	7.04	7.55	2.85
*Ditylum brightwellii*	0.87	1.00	1.71	2.46	2.01	4.30	1.50	0.05	0.58	2.12	1.26	9.09	0.00	2.35	0.95	1.49	7.55	1.31
*Rhizosolenia robusta*	5.76	1.94	1.47	3.77	2.01	0.36	3.50	3.26	3.77	1.27	1.26	0.00	0.00	2.35	4.04	5.03	0.00	2.48
*Chaetoceros denticulatus f.angusta*	1.77	2.49	3.92	1.88	2.26	4.66	1.20	3.73	2.32	0.00	4.40	0.00	0.00	4.82	1.90	2.28	0.00	1.91
*Protoperdinium depressum*	2.86	1.04	1.16	6.38	3.01	0.00	5.69	0.71	2.90	1.27	0.00	0.00	0.00	3.28	5.15	3.50	0.00	0.64
*Nitzschia pungens*	0.15	0.83	0.37	0.00	0.46	15.05	0.40	1.09	0.00	0.00	0.00	0.00	0.00	0.00	0.63	0.67	16.98	1.95
*Thalassionema nitzschioides*	2.60	1.91	2.82	2.75	0.54	1.43	0.20	3.69	0.00	5.08	1.26	0.00	1.22	1.73	0.16	0.00	0.00	1.58
*Ceratium macroceros v.gallicum*	0.79	1.45	2.88	0.29	0.75	0.36	3.70	0.47	4.64	1.27	1.26	0.00	0.00	4.20	3.09	1.26	0.00	1.51
*Coscinodiscus gigas*	0.15	0.10	0.43	0.29	0.13	0.00	0.70	0.14	0.29	0.42	0.00	18.18	2.44	0.43	0.24	0.31	0.00	0.54
*Rhizosolenia bergonii*	0.08	0.83	1.04	0.29	2.46	3.94	0.50	1.37	0.00	0.00	1.26	0.00	0.00	0.80	1.03	1.93	6.60	1.18
Number of species identified	48	49	7	46	26	51	47	54	33	33	27	8	18	54	51	43	15	40
Relative abundance of HABs and potential HABs (%)	17.39	6.78	27.04	9.09	0.61	24.28	17.21	11.74	10.15	22.58	7.94	23.36	22.19	15.09	27.36	13.63	9.27	10.70
Shannon–Wiener Diversity Index (*H*′)	2.73	2.52	2.06	2.74	2.74	2.44	3.37	1.72	3.09	2.96	3.24	3.10	2.98	2.22	2.38	2.52	2.00	1.70
Pielou’s Evenness Index (*J*′)	0.68	0.65	0.60	0.69	0.65	0.67	0.76	0.55	0.72	0.73	0.75	0.74	0.71	0.61	0.64	0.65	0.60	0.54

MR = marine ranching areas; P = peripheral; S = side areas; I = inner bay area; O = outer bay areas; HABs = harmful algae blooms.

**Table 2:** Relative abundance (%) of the top 20 phytoplankton taxa in the Bailong Pearl Bay National Marine Ranching Demonstration Zone and its surrounding areas during summer.

	MR1	MR2	P1	P2	P3	P4	S1	S2	I1	I2	I3	I4	I5	O1	O2	O3	O4	O5
*Rhizosolenia hyalina*	22.84	29.11	2.21	29.88	51.23	29.35	1.50	29.56	1.25	7.73	29.38	21.71	20.57	3.05	10.33	48.03	47.17	24.58
*Rhizosolenia stylisormis*	5.76	4.39	6.24	3.96	4.32	17.65	5.25	15.67	1.58	4.13	4.58	6.36	20.86	2.38	4.54	3.37	8.81	15.28
*Bacteriastrum furcatum*	2.00	3.74	16.27	3.96	4.73	9.39	13.90	7.28	5.51	15.34	7.55	9.50	4.15	9.49	10.30	1.26	2.94	5.06
*Nitzschia pungens*	2.22	0.00	13.23	2.13	0.82	1.23	11.58	0.24	11.88	26.82	1.62	2.44	4.06	21.29	6.89	0.56	0.27	1.33
*Chaetoceros lorenzianus*	3.10	4.72	2.49	3.66	3.50	7.18	2.86	9.91	8.15	6.15	3.23	10.37	10.60	8.48	5.38	2.39	2.87	4.21
*Nitzschia lorenziana*	0.00	1.14	32.01	0.00	0.82	3.03	26.61	0.00	0.00	6.96	0.00	0.61	0.34	5.97	11.72	0.56	3.14	4.72
*Thalassionema frauenfeldii*	4.88	7.97	2.69	6.40	4.12	2.00	2.41	1.35	3.91	1.31	3.77	5.06	2.22	3.09	4.02	5.90	3.34	5.65
*Rhizosolenia imbricata f.imbricata*	6.43	12.85	3.81	2.74	4.32	0.41	3.66	1.59	6.44	3.86	0.81	2.44	1.55	0.60	11.81	3.37	0.82	2.62
*Thalassionema nitzschioides*	1.33	1.30	1.74	3.35	0.00	6.82	3.03	1.47	2.03	3.05	3.23	11.33	6.03	4.49	4.69	3.79	5.12	1.96
*Chaetoceros* spp.	0.00	9.11	1.92	0.00	0.00	0.00	9.00	0.00	34.70	0.00	0.00	0.00	0.00	12.04	0.00	0.00	0.00	0.00
*Bacteriastrum hyalinum v*	10.42	1.30	2.71	3.66	0.82	1.90	1.45	2.33	8.37	4.03	2.43	2.01	0.96	1.48	4.17	2.67	3.28	1.22
*Eucampia zodiacus*	2.00	2.76	0.38	1.83	3.70	2.72	0.11	6.61	1.36	0.00	3.77	2.96	5.74	0.13	2.84	0.00	1.50	5.68
*Pseudosolenia calcar-avis*	0.44	1.14	1.40	0.00	1.65	0.67	1.16	4.83	0.33	0.22	1.89	2.27	3.81	1.07	1.68	2.53	3.69	4.17
*Ceratium tripos*	3.77	1.79	0.02	3.05	1.23	1.44	0.05	0.31	0.03	0.27	8.09	1.22	0.25	0.07	0.03	4.07	2.25	0.18
*Chaetoceros curvisetus*	3.99	0.00	0.00	0.00	0.00	0.00	2.44	0.00	2.96	4.68	0.00	1.74	0.00	8.65	3.79	0.00	0.00	0.00
*Chaetoceros nipponica*	0.00	0.00	0.00	10.67	0.00	1.80	0.00	3.43	0.00	0.00	3.77	0.00	0.50	0.00	0.81	0.00	0.00	4.54
*Stephanopyxis turris*	0.00	0.33	3.71	0.00	0.00	0.00	7.55	0.00	1.21	2.88	0.00	0.00	0.00	3.25	2.14	0.00	0.14	0.00
*Rhizosolenia robusta*	0.67	0.98	0.63	1.22	2.06	0.67	0.34	1.16	0.46	1.09	1.89	2.01	1.30	1.17	1.79	1.40	1.57	0.81
*Guinardia striata*	2.66	0.00	0.00	0.61	0.00	0.92	0.09	1.04	0.00	0.22	0.54	1.05	3.90	0.00	0.00	0.00	0.00	1.96
*Ceratium macroceros v.gallicum*	0.67	0.65	0.04	2.13	0.41	0.77	0.03	0.00	0.02	1.09	5.39	1.48	0.38	0.17	0.03	2.81	2.53	0.07
Number of species identified	38	38	39	35	41	34	47	48	51	28	37	43	43	45	41	37	39	45
Relative abundance of HABs and potential HABs (%)	15.32	36.67	21.02	14.21	13.41	15.39	14.85	10.06	9.67	8.88	8.26	31.29	12.68	10.39	10.72	10.7	17.74	5.37
Shannon–Wiener Diversity Index (*H*′)	3.26	3.66	2.31	3.43	3.43	2.76	2.93	3.37	3.56	2.50	3.37	3.77	3.68	3.28	3.40	3.67	3.37	3.29
Pielou’s Evenness Index (*J*′)	0.66	0.72	0.45	0.69	0.70	0.61	0.56	0.72	0.66	0.55	0.62	0.74	0.81	0.64	0.66	0.73	0.75	0.73

MR = marine ranching areas; P = peripheral; S = side areas; I = inner bay area; O = outer bay areas; HABs = harmful algae blooms.

**Table 3:** Relative abundance (%) of the top 20 phytoplankton taxa in the Bailong Pearl Bay National Marine Ranching Demonstration Zone and its surrounding areas during autumn.

	MR1	MR2	P1	P2	P3	P4	S1	S2	I1	I2	I3	I4	I5	O1	O2	O3	O4	O5
*Chaetoceros lorenzianus*	29.41	21.26	14.26	19.89	22.15	25.23	2.34	30.83	14.02	13.62	10.72	16.04	25.87	17.16	21.05	23.05	19.09	26.81
*Rhizosolenia alata f.gracillima*	11.35	21.43	1.63	2.57	11.10	19.86	3.86	36.27	7.32	8.84	7.34	14.16	13.47	6.82	3.54	18.70	36.22	37.28
*Skeletonema costatum*	10.89	12.18	39.25	23.73	1.84	0.00	25.22	3.24	12.37	14.71	21.27	10.92	8.22	33.60	18.75	3.93	0.00	0.00
*Eucampia zodiacus*	4.41	6.02	3.63	7.92	15.43	7.29	4.23	1.90	8.49	9.76	6.88	5.62	3.44	4.86	18.05	7.28	4.96	2.32
*Bacteriastrum hyalinum*	4.34	7.49	4.30	4.21	10.28	13.61	3.41	2.54	3.05	9.11	0.52	9.31	4.45	2.41	7.09	11.83	8.67	5.25
*Nitzschia pungens*	5.60	2.62	10.45	3.46	3.81	0.00	6.07	1.19	11.38	10.06	3.42	3.52	1.83	4.73	5.55	1.32	0.00	0.11
*Schroderella delicatula*	2.28	0.88	4.68	5.96	2.09	1.54	3.11	2.31	3.61	4.91	6.08	5.95	5.93	8.03	2.45	1.98	0.79	0.21
*Chaetoceros siamense*	3.35	3.02	1.53	1.64	4.13	4.43	1.88	1.95	0.96	1.94	5.69	3.50	3.72	1.74	0.87	2.78	2.68	2.16
*Stephanopyxis palmeriana*	2.21	2.13	1.15	4.23	3.46	3.92	1.12	0.68	0.58	0.57	0.39	0.54	1.43	2.31	3.14	6.95	4.97	3.09
*Chaetoceros affinis*	1.21	1.50	2.55	2.36	1.94	0.35	3.05	0.75	3.20	5.62	4.93	4.54	0.74	0.59	3.31	0.95	0.89	0.47
*Guinardia flaccida*	2.44	2.72	1.30	1.17	3.88	1.70	0.69	2.46	0.18	0.00	0.61	0.73	2.75	1.96	1.41	2.37	5.09	3.87
*Chaetoceros curvisetus*	2.55	0.47	0.32	2.34	1.81	0.23	7.41	0.00	1.50	2.07	3.10	2.69	0.00	0.78	1.48	3.87	0.72	0.33
*Chaetoceros denticulatus*	1.30	1.18	0.07	0.30	1.44	1.70	0.64	1.86	0.63	0.28	0.32	0.32	3.01	1.46	0.36	1.17	2.67	4.93
*Leptocylindrus danicus*	1.11	0.67	0.00	2.57	0.64	0.00	6.82	0.21	3.74	1.55	2.17	1.88	1.28	0.65	0.00	0.89	0.39	0.12
*Chaetoceros diadema*	1.21	0.25	0.82	1.63	0.60	0.94	2.28	0.37	2.42	1.22	3.59	2.64	0.85	1.01	0.62	0.85	0.00	0.40
*Rhizosolenia alata*	1.46	1.82	0.30	1.01	0.82	0.84	0.66	2.04	0.51	1.02	0.84	0.70	3.49	0.92	0.24	1.46	0.61	1.09
*Chaetoceros rostratus*	0.50	1.02	0.80	0.63	1.04	0.94	1.95	1.48	1.75	1.44	1.21	0.47	1.13	1.48	0.71	0.68	0.50	1.20
*Bacteriastrum furcatum*	0.61	2.49	0.32	0.59	0.82	1.11	1.16	0.52	0.83	1.07	1.43	1.94	1.79	0.63	0.62	0.65	0.46	0.99
*Chaetoceros paradoxus*	0.52	1.17	1.22	0.75	0.25	0.46	2.16	0.52	1.63	1.91	1.21	1.08	1.19	0.71	0.65	0.43	0.00	0.00
Number of species identified	59	63	52	52	58	65	59	55	60	46	55	53	61	61	58	59	50	59
Relative abundance of HABs and potential HABs (%)	28.87	32.63	32.94	22.15	11.37	42.65	54.15	32.28	10.21	3.59	6.48	40.46	30.12	10.55	2.87	2.32	14.57	20.49
Shannon–Wiener Diversity Index (*H*′)	4.32	4.24	4.38	4.13	3.92	3.61	4.43	4.38	3.78	3.24	3.64	2.72	1.75	4.13	3.92	3.61	5.71	4.63
Pielou’s Evenness Index (*J*′)	0.80	0.77	0.82	0.81	0.72	0.80	0.83	0.82	0.79	0.70	0.82	0.97	0.45	0.81	0.72	0.80	0.82	0.85

MR = marine ranching areas; P = peripheral; S = side areas; I = inner bay area; O = outer bay areas; HABs = harmful algae blooms.

**Table 4:** Relative abundance (%) of the top 20 phytoplankton taxa in the Bailong Pearl Bay National Marine Ranching Demonstration Zone and its surrounding areas during winter.

	MR1	MR2	P1	P2	P3	P4	S1	S2	I1	I2	I3	I4	I5	O1	O2	O3	O4	O5
*Nitzschia pungens*	94.44	96.75	75.89	97.92	82.14	62.53	48.48	92.88	45.39	70.87	94.22	88.70	78.77	90.05	94.12	93.98	91.20	50.00
*Nitzschia paradoxa*	0.00	0.00	0.00	0.00	0.14	0.00	16.16	0.00	31.91	0.00	0.00	0.00	0.00	0.00	0.00	0.16	0.00	0.00
*Rhizosolenia alata f. gracillima*	0.43	0.14	16.07	0.07	0.81	6.05	0.00	0.09	1.42	11.51	0.00	0.00	0.00	0.00	0.22	0.00	0.62	0.00
*Rhizosolenia hyalina*	0.65	0.41	1.65	0.61	2.30	1.44	2.02	1.95	0.00	1.76	1.62	2.58	3.87	2.70	1.20	0.69	1.00	0.00
*Pseudosolenia calcaravis*	0.42	0.19	1.03	0.10	1.44	1.44	6.06	0.39	0.71	2.42	0.00	0.30	0.50	0.19	1.09	0.79	0.95	3.95
*Chaetoceros constrictus*	0.00	0.35	0.00	0.00	1.34	12.71	0.00	0.32	0.00	0.00	0.00	0.00	1.58	0.00	0.25	0.00	0.15	0.00
*Rhizosolenia alata*	0.91	0.11	1.38	0.03	1.80	1.49	0.00	0.20	2.13	0.00	0.00	0.00	2.79	0.38	1.24	0.29	1.47	0.00
*Chaetoceros curvisetus*	0.92	0.45	0.36	0.00	1.36	0.00	0.00	0.00	0.00	0.36	2.04	3.75	2.04	0.00	0.00	2.01	0.00	0.00
*Stephanopyxis palmeriana*	0.04	0.12	0.00	0.07	0.12	0.21	0.00	0.42	0.00	0.12	0.39	0.41	0.37	0.09	0.01	0.06	0.17	10.53
*Thalassionema nitzschioides*	0.03	0.04	0.09	0.00	0.00	0.31	2.02	0.05	1.42	0.24	0.00	0.00	0.17	0.00	0.00	0.13	0.49	7.89
*Climacodium biconcavum*	0.60	0.29	0.49	0.14	1.56	0.56	2.02	0.31	1.42	0.36	0.41	0.30	0.83	2.23	0.57	0.30	0.35	0.00
*Chaetoceros lorenzianus*	0.00	0.04	0.27	0.00	0.34	1.95	0.00	0.03	0.00	0.48	0.00	0.00	0.00	0.00	0.05	0.00	0.23	9.21
*Skeletonema costatum*	0.00	0.00	0.00	0.00	0.00	2.51	0.00	0.00	0.00	0.00	0.00	0.00	0.00	0.00	0.00	0.00	0.00	7.89
*Coscinodiscus asteromphalus*	0.02	0.14	0.04	0.17	0.02	0.00	6.06	0.02	2.13	0.12	0.15	0.10	0.08	0.43	0.01	0.03	0.04	0.00
*Coscinodiscus gigas*	0.03	0.07	0.04	0.12	0.05	0.21	3.03	0.03	0.00	0.06	0.11	0.25	0.04	0.00	0.02	0.04	0.14	5.26
*Chaetoceros rostratus*	0.15	0.00	0.00	0.00	3.60	1.95	0.00	1.04	0.00	0.73	0.00	0.00	1.50	0.00	0.16	0.25	0.09	0.00
*Chaetoceros affinis*	0.00	0.00	0.00	0.00	0.00	0.00	4.04	0.00	0.00	0.85	0.00	0.00	3.48	0.00	0.00	0.00	0.00	0.00
*Rhizosolenia robusta*	0.07	0.06	0.00	0.03	0.16	0.10	4.04	0.01	0.00	0.18	0.09	0.15	0.17	0.28	0.07	0.08	0.08	0.00
*Rhizosolenia alata f. indica*	0.00	0.00	0.09	0.00	0.00	0.00	0.00	0.00	0.00	4.91	0.04	0.00	0.00	0.00	0.00	0.00	0.04	0.00
*Stephanopyxis turris*	0.14	0.04	0.18	0.00	0.33	0.10	0.00	0.67	0.00	0.00	0.00	1.22	1.17	0.00	0.20	0.25	0.67	0.00
Number of species identified	41	37	35	26	29	38	15	40	19	37	22	23	39	24	34	34	40	11
Relative abundance of HABs and potential HABs (%)	47.52	72.2	96.34	92.7	84.43	52.53	76.38	98.16	83.53	65.09	92.88	90.52	94.13	96.04	91.2	57.89	95.41	97.33
Shannon–Wiener Diversity Index (*H*′)	0.53	0.35	1.34	0.23	1.36	2.26	2.67	0.63	2.42	1.84	0.49	0.87	1.56	0.82	0.52	0.54	0.79	2.47
Pielou’s Evenness Index (*J*′)	0.10	0.07	0.26	0.05	0.26	0.47	0.68	0.12	0.57	0.35	0.11	0.19	0.29	0.18	0.10	0.11	0.15	0.72

MR = marine ranching areas; P = peripheral; S = side areas; I = inner bay area; O = outer bay areas; HABs = harmful algae blooms.

**Table 5:** Spearman’s correlation coefficients among environmental parameters, inorganic nutrients, and phytoplankton abundance. Bold indicating significant difference at *p* < 0.05.

	Phytoplankton	HABs	Temperature	Salinity	pH	DO	Nitrite	Nitrate	Ammonium	Phosphate
Phytoplankton		0.43	**0.31**	−0.43	**0.31**	**−0.26**	**0.20**	**0.40**	**−0.19**	**0.37**
HABs			**−0.49**	−0.13	0.19	**0.52**	−0.69	**0.50**	−0.10	**−0.30**
Temperature				**−0.37**	−0.15	**−0.81**	**0.60**	**−0.28**	−0.11	**0.80**
Salinity					**0.45**	**0.25**	**−0.66**	−0.20	−0.10	**−0.62**
pH						0.18	**−0.32**	0.17	−0.17	**−0.24**
DO							**−0.39**	**0.34**	0.30	**−0.64**
Nitrite								0.88	**−0.15**	**0.78**
NItrate									−0.02	**−0.37**
Ammonium										0.31
Phosphate										

## Data Availability

The data supporting the findings of this study are available from the corresponding authors upon reasonable request.

## Supplementary Materials

The following supporting information can be downloaded at https://www.mdpi.com/article/10.3390/biology15060477/s1, Table S1: Overall spatial means of the relative abundance (%) of top 20 phytoplankton taxa in the Bailong Pearl Bay National Marine Ranching Demonstration Zone and its surrounding areas during spring; Table S2: Overall spatial means of the relative abundance (%) of top 20 phytoplankton taxa in the Bailong Pearl Bay National Marine Ranching Demonstration Zone and its surrounding areas during summer; Table S3: Overall spatial means of the relative abundance (%) of top 20 phytoplankton taxa in the Bailong Pearl Bay National Marine Ranching Demonstration Zone and its surrounding areas during autumn; Table S4: Overall spatial means of the relative abundance (%) of top 20 phytoplankton taxa in the Bailong Pearl Bay National Marine Ranching Demonstration Zone and its surrounding areas during winter.
