# Supplementary material for: Effects of Marine Ranching on Phytoplankton Community: A Case Study in the Bailong Pearl Bay National Marine Ranching Demonstration Zone, China
Source: Biology (Basel). 2026 Mar 16;15(6):477. doi: 10.3390/biology15060477 (PMC13024520; doi:10.3390/biology15060477)
Supplement: Supplementary file 1 [file biology-15-00477-s001.zip › biology-4189704-supplementary.pdf]

Supplementary Table S1: Overall spatial means of the relative abundance (%) of top 20 phytoplankton taxa in the Bailong Pearl Bay National Marine Ranching Demonstration Zone and its surrounding areas during Spring

	MR	P	S	I	O
Coscinodiscus granii	7.20± 4.10	6.27± 5.34	8.53± 6.45	12.13± 9.14	6.13± 4.81
Thalassiosira subtilis	3.32± 1.77	2.84± 3.51	7.33± 6.41	18.96± 29.45	4.74± 5.47
Bacteriastrum hyalinum v	11.81± 1.67	12.86± 11.14	6.87± 5.19	4.04± 4.70	5.44± 4.18
Thalassionema frauenfeldii	10.36± 1.12	14.22± 7.42	5.12± 5.40	1.44± 1.54	8.07± 5.41
Chaetoceros coarctatus	3.49± 2.09	5.40± 3.55	11.64± 16.46	9.77± 9.38	6.46± 6.27
Chaetoceros lorenzianus	8.00± 2.35	5.29± 2.14	5.58± 0.40	9.95± 12.40	5.64± 2.53
Stephanopyxis palmeriana	4.15± 0.91	2.74± 2.18	6.56± 6.30	3.18± 3.40	4.88± 5.07
Guinardia flaccida	6.63± 2.82	6.09± 4.52	2.74± 1.75	0.51± 0.69	4.87± 6.32
Skeletonema costatum	2.84± 0.68	4.00± 2.01	2.88± 2.72	3.77± 8.44	4.11± 1.64
Hemiaulus sinensis	8.25± 5.43	3.24± 2.17	2.07± 0.33	0.67± 1.10	5.12± 4.22
Rhizosolenia hyalina	4.03± 0.60	2.84± 0.10	3.85± 0.64	0.35± 0.78	5.12± 3.06
Ditylum brightwellii	0.94± 0.09	2.62± 1.16	0.78± 1.03	2.61± 3.71	2.73± 2.74
Rhizosolenia robusta	3.85± 2.70	1.90± 1.42	3.38± 0.17	1.26± 1.54	2.78± 1.91
Chaetoceros denticulatus f.angusta	2.13± 0.51	3.18± 1.33	2.47± 1.79	1.34± 1.98	2.18± 1.72
Protoperdinium depressum	1.95± 1.29	2.64± 2.79	3.20± 3.52	0.83± 1.28	2.51± 2.14
Nitzschia pungens	0.49± 0.48	3.97± 7.39	0.75± 0.49	0.00	4.05± 7.26
Thalassionema	2.26± 0.49	1.89± 1.10	1.95± 2.47	1.51± 2.09	0.69± 0.88

nitzschioides					
Ceratium macroceros v.gallicum	1.12± 0.47	1.07± 1.22	2.09± 2.28	1.43± 1.90	2.01± 1.64
Coscinodiscus gigas	0.13± 0.04	0.21± 0.19	0.42± 0.40	4.27± 7.84	0.30± 0.21
Rhizosolenia bergonii	0.46± 0.53	1.93± 1.61	0.94± 0.62	0.25± 0.56	2.31± 2.44
Diversity	2.63± 0.15	2.50± 0.32	2.55± 1.17	3.07± 0.11	2.16± 0.32
Eveness	0.67± 0.02	1.15± 1.01	0.66± 0.15	0.73± 0.02	0.61± 0.04

MR= marine ranching areas (n=2); P= peripheral (n=4); S= side areas (n=2); I= inner bay area (n=5); O= outer bay areas (n=5)

Supplementary Table S2: Overall spatial means of the relative abundance (%) of top 20 phytoplankton taxa in the Bailong Pearl Bay National Marine Ranching Demonstration Zone and its surrounding areas during Summer

	MR	P	S	I	O
Rhizosolenia hyalina	25.98± 4.43	28.17± 20.08	15.53± 19.84	16.13± 11.38	26.63± 20.65
Rhizosolenia stylisormis	5.08± 0.97	8.04± 6.48	10.46± 7.37	7.50± 7.66	6.88± 5.30
Bacteriastrum furcatum	2.87± 1.23	8.59± 5.66	10.59± 4.68	8.41± 4.37	5.81± 3.98
Nitzschia pungens	1.11± 1.57	4.35± 5.94	5.91± 8.02	9.36± 10.57	6.07± 8.93
Chaetoceros lorenzianus	3.91± 1.15	4.21± 2.05	6.39± 4.99	7.70± 3.09	4.67± 2.43
Nitzschia lorenziana	0.57± 0.81	8.97± 15.42	13.31± 18.82	1.58± 3.02	5.22± 4.16
Thalassionema frauenfeldii	6.43± 2.18	3.80± 1.94	1.88± 0.75	3.25± 1.48	4.40± 1.30
Rhizosolenia imbricata f.imbricata	9.64± 4.54	2.82± 1.74	2.63± 1.46	3.02± 2.22	3.84± 4.61
Thalassionema nitzschioides	1.32± 0.02	2.98± 2.90	2.25± 1.10	5.13± 3.77	4.01± 1.24
Chaetoceros spp	4.56± 6.44	0.48± 0.96	4.50± 6.36	6.94± 15.52	2.41± 5.38
Bacteriastrum hyalinum v	5.86± 6.45	2.27± 1.21	1.89± 0.62	3.56± 2.91	2.56± 1.23
Eucampia zodiacus	2.38± 0.54	2.16± 1.41	3.36± 4.60	2.77± 2.21	2.03± 2.34
Pseudosolenia calcar-avis	0.79± 0.49	0.93± 0.75	3.00± 2.60	1.70± 1.49	2.63± 1.31
Ceratium tripos	2.78± 1.40	1.44± 1.25	0.18± 0.18	1.97± 3.45	1.32± 1.80
Chaetoceros curvisetus	2.00± 2.82	0.00	1.22± 1.73	1.88± 2.01	2.49± 3.82
Chaetoceros nipponica	0.00	3.12± 5.11	1.72± 2.43	0.85± 1.64	1.07± 1.97
Stephanopyxis turris	0.17± 0.23	0.93± 1.86	3.78± 5.34	0.82± 1.27	1.11± 1.50

Rhizosolenia robusta	0.83± 0.22	1.15± 0.67	0.75± 0.58	1.35± 0.63	1.35± 0.38
Guinardia striata	1.33± 1.88	0.38± 0.46	0.57± 0.67	1.14± 1.59	0.39± 0.88
Ceratium macroceros v.gallicum	0.66± 0.01	0.84± 0.91	0.02± 0.02	1.67± 2.16	1.12± 1.42
Diversity	3.46± 0.28	2.98± 0.55	3.15± 0.31	3.38± 0.51	3.40± 0.16
Eveness	0.69± 0.04	0.61± 0.12	0.64± 0.11	0.68± 0.10	0.70± 0.05

MR= marine ranching areas (n=2); P= peripheral (n=4); S= side areas (n=2); I= inner bay area (n=5); O= outer bay areas (n=5)

Supplementary Table S3: Overall spatial means of the relative abundance (%) of top 20 phytoplankton taxa in the Bailong Pearl Bay National Marine Ranching Demonstration Zone and its surrounding areas during Autumn

	MR	P	S	I	O
Chaetoceros lorenzianus	25.34± 5.76	20.38± 4.63	16.59± 20.15	16.05± 5.81	21.43± 3.72
Rhizosolenia alata f.gracillima	16.39± 7.13	8.79± 8.52	20.07± 22.92	10.23± 3.34	20.51± 15.86
Skeletonema costatum	11.54± 0.91	16.21± 18.77	14.23± 15.54	13.50± 4.94	11.26± 14.68
Eucampia zodiacus	5.22± 1.14	8.57± 4.95	3.07± 1.65	6.84± 2.46	7.49± 6.16
Bacteriastrum hyalinum v	5.92± 2.23	8.10± 4.64	2.98± 0.62	5.29± 3.85	7.05± 3.54
Nitzschia pungens	4.11± 2.11	4.43± 4.37	3.63± 3.45	6.04± 4.35	2.34± 2.62
Schroderella delicatula	1.58± 0.99	3.57± 2.10	2.71± 0.57	5.30± 1.05	2.69± 3.12
Chaetoceros siamense	3.19± 0.23	2.93± 1.56	1.92± 0.05	3.16± 1.81	2.05± 0.78
Stephanopyxis palmeriana	2.17± 0.06	3.19± 1.40	0.90± 0.31	0.70± 0.41	4.09± 1.87
Chaetoceros affinis	1.36± 0.21	1.80± 1.40	1.90± 1.63	3.81± 1.93	1.24± 1.17
Guinardia flaccida	2.58± 0.20	2.01± 1.27	1.58± 1.25	0.85± 1.10	2.94± 1.51
Chaetoceros curvisetus	1.51± 1.47	1.18± 1.06	3.71± 5.24	1.87± 1.21	1.44± 1.42
Chaetoceros denticulatus	1.24± 0.08	0.88± 0.81	1.25± 0.86	0.91± 1.18	2.12± 1.78
Leptocylindrus danicus	0.89± 0.31	0.80± 1.22	3.52± 4.67	2.12± 0.96	0.41± 0.37
Chaetoceros diadema	0.73± 0.68	1.00± 0.44	1.33± 1.35	2.14± 1.11	0.58± 0.40
Rhizosolenia alata	1.64± 0.25	0.74± 0.31	1.35± 0.98	1.31± 1.23	0.86± 0.46
Chaetoceros rostratus	0.76± 0.37	0.85± 0.18	1.72± 0.33	1.20± 0.47	0.91± 0.41
Bacteriastrum furcatum	1.55± 1.33	0.71± 0.34	0.84± 0.45	1.41± 0.47	0.67± 0.19
Chaetoceros paradoxus	0.85± 0.46	0.67± 0.42	1.34± 1.16	1.40± 0.35	0.36± 0.34

Diversity	4.28± 0.06	4.01± 0.33	4.41± 0.04	3.03± 0.82	4.40± 0.82
Eveness	0.79± 0.02	0.79± 0.05	0.83± 0.01	0.75± 0.19	0.84± 0.12

MR= marine ranching areas (n=2); P= peripheral (n=4); S= side areas (n=2); I= inner bay area (n=5); O= outer bay areas (n=5)

Supplementary Table S4: Overall spatial means of the relative abundance (%) of top 20 phytoplankton taxa in the Bailong Pearl Bay National Marine Ranching Demonstration Zone and its surrounding areas during Winter

	MR	P	S	I	O
Nitzschia pungens	95.60± 1.63	79.62± 14.69	70.68± 31.40	75.59± 19.13	83.87± 19.02
Nitzschia paradoxa	0	0.04± 0.07	8.08± 11.43	6.38± 14.27	0.03± 0.07
Rhizosolenia alata f. gracillima	0.29± 0.21	5.75± 7.38	0.05± 0.06	2.59± 5.03	0.17± 0.27
Rhizosolenia hyalina	0.53± 0.17	1.50± 0.70	1.99± 0.05	1.97± 5.03	1.12± 0.99
Pseudosolenia calcaravis	0.31± 0.16	1.00± 0.63	3.23± 4.01	0.79± 0.95	1.39± 1.47
Chaetoceros constrictus	0.18± 0.25	3.51± 6.16	0.16± 0.23	0.32± 0.71	0.08± 0.12
Rhizosolenia alata	0.51± 0.57	1.18± 0.78	0.10± 0.14	0.98± 1.37	0.68± 0.64
Chaetoceros curvisetus	0.69± 0.33	0.43± 0.64	0	1.64± 1.51	0.40± 0.90
Stephanopyxis palmeriana	0.08± 0.06	0.10± 0.09	0.21± 0.30	0.26± 0.19	2.17± 4.67
Thalassionema nitzschioides	0.04± 0.01	0.10± 0.15	1.04± 1.39	0.37± 0.60	1.70± 3.47
Climacodium biconcavum	0.45± 0.22	0.69± 0.61	1.17± 1.21	0.66± 0.47	0.69± 0.88
Chaetoceros lorenzianus	0.02± 0.03	0.64± 0.89	0.02± 0.02	0.10± 0.21	1.90± 4.09
Skeletonema costatum	0	0.63± 1.26	0	0	1.58± 3.53
Coscinodiscus asteromphalus	0.08± 0.08	0.06± 0.08	3.04± 4.27	0.52± 0.90	0.10± 0.18
Coscinodiscus gigas	0.05± 0.03	0.11± 0.08	1.53± 2.12	0.09± 0.10	1.09± 2.33
Chaetoceros rostratus	0.08± 0.11	1.39± 1.74	0.52± 0.74	0.45± 0.67	0.10± 0.11
Chaetoceros affinis	0	0	2.02± 2.86	0.87± 1.51	0

Rhizosolenia robusta	0.07± 0.01	0.07± 0.07	2.03± 2.85	0.12± 0.07	0.10± 0.10
Rhizosolenia alata f. indica	0	0.02± 0.05	0	0.99± 2.19	0.01± 0.02
Stephanopyxis turris	0.09± 0.07	0.15± 0.14	0.34± 0.47	0.48± 0.65	0.22± 0.27
Diversity	0.44± 0.13	1.30± 0.83	1.65± 1.44	1.44± 0.77	1.03± 0.82
Eveness	0.09± 0.02	0.26± 0.17	0.40± 0.40	0.30± 0.18	0.25± 0.26

MR= marine ranching areas (n=2); P= peripheral (n=4); S= side areas (n=2); I= inner bay area (n=5); O= outer bay areas (n=5)
